# Dual-Mode Optical Detection of Sulfide Ions Using Copper-Anchored Nitrogen-Doped Graphene Quantum Dot Nanozymes

**DOI:** 10.3390/bios15080528

**Published:** 2025-08-13

**Authors:** Van Anh Ngoc Nguyen, Trung Hieu Vu, Phuong Thy Nguyen, Moon Il Kim

**Affiliations:** Department of BioNano Technology, Gachon University, 1342 Seongnamdae-ro, Sujeong-gu, Seongnam 13120, Republic of Korea; vananh.ngocng@gmail.com (V.A.N.N.); hieu.vutrung24596@gmail.com (T.H.V.); nnphuongthy18@gmail.com (P.T.N.)

**Keywords:** nanozymes, graphene quantum dot, sulfide ion detection, dual-mode sensing

## Abstract

We present a dual-mode optical sensing strategy for selective and sensitive detection of sulfide ions (S^2−^), employing copper-anchored nitrogen-doped graphene quantum dots (Cu@N-GQDs) as bifunctional nanozymes. The Cu@N-GQDs were synthesized via citric acid pyrolysis in the presence of ammonium hydroxide (serving as both nitrogen source and reductant) and copper chloride, leading to uniform incorporation of copper oxide species onto the N-GQD surface. The resulting nanohybrids exhibit two synergistic functionalities: intrinsic fluorescence comparable to pristine N-GQDs, and significantly enhanced peroxidase-like catalytic activity attributed to the anchored copper species. Upon interaction with sulfide ions, the system undergoes a dual-optical response: (i) fluorescence quenching via Cu-S complexation, and (ii) inhibition of peroxidase-like activity due to the deactivation of Cu catalytic centers via the interaction with S^2−^. This dual-signal strategy enables sensitive quantification of S^2−^, achieving detection limits of 0.5 µM (fluorescence) and 3.5 µM (colorimetry). The sensor demonstrates excellent selectivity over competing substances and high reliability and precision in real tap water samples. These findings highlight the potential of Cu@N-GQDs as robust, bifunctional, and field-deployable nanozyme probes for environmental and biomedical sulfide ion monitoring.

## 1. Introduction

Sulfide ions (S^2−^), along with their conjugate species hydrogen sulfide (H_2_S) and bisulfide (HS^−^), are widely distributed across both natural and anthropogenic systems and play multifaceted roles in environmental, biological, and industrial contexts [1]. These sulfur-containing species are commonly generated through microbial sulfate reduction in anaerobic conditions, as found in aquatic sediments, sewage treatment facilities, and biological systems such as the human gut microbiota [2,3,4]. In parallel, industrial processes including petroleum refining, pulp and paper manufacturing, textile dyeing, leather tanning, and the desulfurization of fossil fuels release substantial quantities of sulfide ions into the environment [5]. From an industrial perspective, sectors that generate sulfur-containing byproducts—including S^2−^—as part of their core production processes operate on a vast global scale and have substantial economic and environmental footprints. For example, the global pulp and paper industry was valued at approximately USD 354.39 billion in 2023 and is projected to surpass USD 373.49 billion by 2024 [6]. Similarly, the petroleum refining industry exceeded USD 600 billion in market value in 2023 alone [7]. These industries, while essential to the global economy and infrastructure, are also among the primary contributors to sulfide emissions. In biological settings, H_2_S functions as a signaling molecule and modulator of physiological processes, particularly in vasodilation, cytoprotection, and neuromodulation [8,9]. However, an excess of sulfide—either in gaseous or ionic form—can be highly toxic, contributing to mitochondrial dysfunction, cellular oxidative stress, and respiratory inhibition [10]. From an environmental standpoint, elevated levels of S^2−^ in aquatic ecosystems can induce eutrophication, corrosion of infrastructure, and foul odor generation, thereby necessitating its timely and accurate monitoring. Hence, the precise quantification of sulfide ions is of considerable importance for ecological safety, industrial hygiene, and human health management.

Numerous analytical techniques have been developed to detect sulfide ions, ranging from classical wet chemical methods to modern instrumental techniques. Gas chromatography and ion-selective electrodes offer accurate quantification, while iodometric titration remains a reference method in many regulatory protocols [11,12]. Electrochemical sensors, including amperometric and potentiometric devices, have shown promising sensitivity and relatively low detection limits [12]. However, despite their accuracy, most of these approaches require expensive instrumentation, skilled personnel, and labor-intensive sample preparation steps, making them less ideal for rapid, on-site measurements or real-time environmental monitoring. As a result, optical sensing methods have gained increasing attention due to their inherent advantages, such as operational simplicity, rapid response time, low cost, and visual interpretability. Among these, colorimetric and fluorescence-based assays are particularly attractive owing to their capability for naked-eye or spectrophotometric detection without the need for sophisticated equipment [13,14].

Despite the progress made, a critical limitation remains: most current optical sensors rely on a single-mode output. While effective under ideal laboratory conditions, single-mode sensors are often susceptible to environmental noise, matrix effects, and signal instability when deployed in real-world settings. For instance, colorimetric signals can be obscured by naturally colored samples or turbidity, while fluorescence intensity can be affected by photobleaching or background fluorescence [15,16]. This has driven recent interest in developing dual-mode sensors that combine orthogonal detection mechanisms to improve reliability and reduce false positives. Along these lines, a supramolecular probe was reported to exhibit fluorescent bicolor signals for barium ion detection [17]. Uniquely designed quinoxalinone-based probe exhibited fluorescent signals in both liquid and solid states, enabling excellent dual-state sensing for nerve agents [18]. Likewise, a dual-output system not only provides internal validation but also enhances analytical confidence, especially when dealing with complex samples.

Recent advancements in nanomaterials have provided powerful platforms for the development of optical sensors with high efficacy for sulfide ion detection. Carbon dot-based fluorescent probes quenched by metal ions such as Hg^2+^ or Ag^+^ exhibited fluorescence recovery upon sulfide-induced metal precipitation [19]. Graphene quantum dots (GQDs) coordinated with Cu^2+^ showed aggregation-mediated fluorescence suppression, which is reversed upon sulfide exposure [20]. Colorimetric sensors based on plasmonic nanoparticles, including Au–Ag core-shell nanostructures, provide visual readouts through sulfide-induced formation of Ag_2_S [21]. Moreover, nanozyme-based approaches—mimicking enzymatic catalysis—have demonstrated high sensitivity for sulfide via activity inhibition, as exemplified in our previous work using catalase-like polydopamine-coated Co_3_O_4_ nanoparticles [22]. Despite these advances, the dependence on single-signal mechanisms or complex probe activation processes still limits robustness and applicability in real-world conditions.

In this study, we report a dual-mode (fluorometric/colorimetric) optical sensor based on copper-anchored nitrogen-doped graphene quantum dots (Cu@N-GQDs) that integrates two orthogonal transduction mechanisms into a single nanoplatform. The Cu@N-GQDs maintain the native fluorescence of N-GQDs while incorporating catalytically active Cu_2_O moieties that mimic peroxidase activity. In the absence of sulfide, Cu@N-GQDs catalyze the H_2_O_2_-mediated oxidation of 3,3′,5,5′-tetramethylbenzidine (TMB), yielding a blue colorimetric signal, while also emitting strong fluorescence. Upon exposure to sulfide ions, the formation of Cu–S complexes simultaneously quenches fluorescence and inhibits the peroxidase-like activity. This synergistic signal suppression allows for dual-modality detection of S^2−^ with high sensitivity (detection limits: 0.5 µM fluorescence, 3.5 µM colorimetry) and excellent selectivity. The platform also demonstrated high recovery and precision in real tap water, suggesting its practical applicability in both environmental and biomedical contexts. Our Cu@N-GQD-based system thus provides an efficient, self-validating tool for accurate sulfide ion detection.

## 2. Experimental Section

### 2.1. Reagents and Materials

Copper (II) chloride dihydrate (CuCl_2_·2H_2_O), citric acid, ammonium hydroxide, TMB, and dialysis tubing were purchased from Sigma-Aldrich (St. Louis, MO, USA). Hydrogen peroxide and sodium sulfide (Na_2_S) were obtained from Samchun Chemical (Seoul, Korea). All solutions were prepared in deionized water purified using a Milli-Q Purification System. (Millipore, Billerica, MA, USA).

### 2.2. Synthesis and Characterization of Cu@N-GQDs

Cu@N-GQDs were synthesized via a one-pot hydrothermal carbonization process using citric acid (C_6_H_8_O_7_), ammonium hydroxide, and copper chloride, with modifications to a previously reported protocol (Figure S1) [23]. Briefly, 40 mL of citric acid solution (10 mg/mL) was mixed with 8 mL of ammonium hydroxide (25 wt%) and copper (II) chloride dihydrate solution (10 mg/mL). The resulting mixture was transferred to a Teflon-lined stainless-steel autoclave and heated at 200 °C for 4 h. After naturally cooling to room temperature (RT), the reaction mixture was centrifuged at 8000× *g* for 15 min to remove insoluble residues. The supernatant containing Cu@N-GQDs was collected and subjected to dialysis (MWCO: 2000 Da, Sigma-Aldrich) against deionized water for 24 h to eliminate small-molecule byproducts. The dialyzed product was then lyophilized to yield a brown powder. N-GQDs were prepared in parallel under identical conditions, excluding the addition of copper precursor.

Morphological characterization of Cu@N-GQDs and N-GQDs was carried out using transmission electron microscopy at an acceleration voltage of 300 kV (TEM, FEI Tecnai, Hillsboro, OR, USA). TEM samples were prepared by depositing 5 μL of the dispersed sample onto a carbon-coated copper grid (Electron Microscopy Sciences, Hatfield, PA, USA), followed by overnight drying. Elemental distribution was analyzed by energy-dispersive X-ray spectroscopy (EDS, Bruker, Berlin, Germany). Structural and chemical information was obtained using Fourier transform infrared spectroscopy (FT-IR, JASCO FT/IR-4600, Tokyo, Japan), powder X-ray diffraction (XRD, Rigaku D/MAX-2500, Tokyo, Japan), and X-ray photoelectron spectroscopy (XPS, Gemini, Molecular Devices, San Jose, CA, USA). Dynamic light scattering (DLS) analysis was performed using a Zetasizer (Malvern Instruments, Malvern, Worcestershire, UK) to evaluate the hydrodynamic size distribution of the Cu@N-GQDs.

### 2.3. Evaluation of Fluorescent and Peroxidase-Mimetic Properties

All measurements were performed in triplicate (*n* = 3), representing three independent experimental replicates for each assay, including the assessment of fluorescent and peroxidase-like properties, as well as fluorometric/colorimetric detection of sulfide ions.

The intrinsic fluorescence of Cu@N-GQDs was measured at a concentration of 100 μg/mL in 50 mM HEPES buffer (pH 5.0) using a microplate reader (Synergy H1, BioTek, Winooski, VT, USA) serviced by the Center for Bionano Materials Research at Gachon University (Seongnam, Republic of Korea). Excitation was set at 345 nm, and emission spectra were recorded from 380 to 600 nm. Fluorescent images were acquired using a fluorescence imaging system (Kodak, Tokyo, Japan).

Peroxidase-like activity was evaluated by monitoring the catalytic oxidation of TMB in the presence of hydrogen peroxide (H_2_O_2_). The reaction mixture consisted of Cu@N-GQDs (100 μg/mL), TMB (0.5 mM), and H_2_O_2_ (10 mM) in 50 mM HEPES buffer (pH 5.0). After incubation at RT for 15 min, the absorbance at 652 nm was measured. Detailed assay conditions for measuring the peroxidase-like activity of Cu@N-GQDs are summarized in Table S1. Control experiments to evaluate oxidase-like activity were performed under identical conditions but without H_2_O_2_. The effects of assay conditions, including nanoparticle concentration, incubation time, pH, and temperature, on the peroxidase-like activity of Cu@N-GQDs were evaluated in ranges of parameters. The stability profiles of Cu@N-GQDs and free horseradish peroxidase (HRP) were assessed by incubating each sample for 5 h in sodium acetate buffer under varying conditions: pH 3.0–9.0 at RT and temperatures ranging from 4 to 80 °C at pH 5.0. Enzymatic activity was evaluated by measuring the colorimetric signal of TMB oxidation under standard assay conditions. Relative activity (%) was calculated as the ratio of residual activity after treatment to the initial activity prior to incubation.

Steady-state kinetic assays were conducted by varying TMB or H_2_O_2_ concentrations while maintaining the other at a constant level. Kinetic parameters, including the Michaelis-Menten constant (K_m_) and maximum velocity (V_max_), were calculated using Lineweaver-Burk plots according to the Michaelis-Menten equation, v = V_max_ × [S]/(K_m_ + [S]), where v is the initial velocity and [S] is the concentration of varying substrate TMB or H_2_O_2_.

### 2.4. Dual-Mode Optical Detection of Sulfide Ions Using Cu@N-GQDs

For fluorescence-based detection of sulfide ions, Cu@N-GQDs (100 μg/mL) were incubated with various concentrations of Na_2_S (0–600 μM) in 50 mM HEPES buffer (pH 5.0) at RT for 15 min. The fluorescence intensity at 445 nm (excitation wavelength = 345 nm) was recorded, and signal intensity was expressed as ΔF = F_0_ − F, where F_0_ and F are the fluorescence intensities without and with S^2−^, respectively. For colorimetric detection, identical incubation with Na_2_S was followed by the addition of H_2_O_2_ (10 mM) and TMB (0.5 mM). After 15 min of further incubation, absorbance at 652 nm was measured to quantify sulfide-induced inhibition of catalytic activity.

For selectivity evaluation, the fluorometric and colorimetric responses to sulfide ions (100 µM) were defined as 100%, and the responses from potential interfering substances (2 mM) were expressed as relative activity (%) with respect to the sulfide signal. The limit of detection (LOD) was determined using the formula LOD = 3 × SD_blank/S, where SD_blank is the standard deviation of the blank measurements and S is the slope of the calibration curve.

To validate practical applicability, tap water samples were collected from the laboratory and filtered through 0.45 μm syringe membranes. The filtered water was spiked with defined concentrations of Na_2_S (25, 50, 100, and 150 μM) and analyzed as described above. The recovery rate (%) was calculated as (measured concentration/spiked concentration) × 100, and assay precision was assessed by calculating the coefficient of variation (CV = SD/mean × 100) from three independent replicates.

## 3. Results and Discussion

### 3.1. Synthesis of Bifunctional Cu@N-GQDs

**Figure 1:**
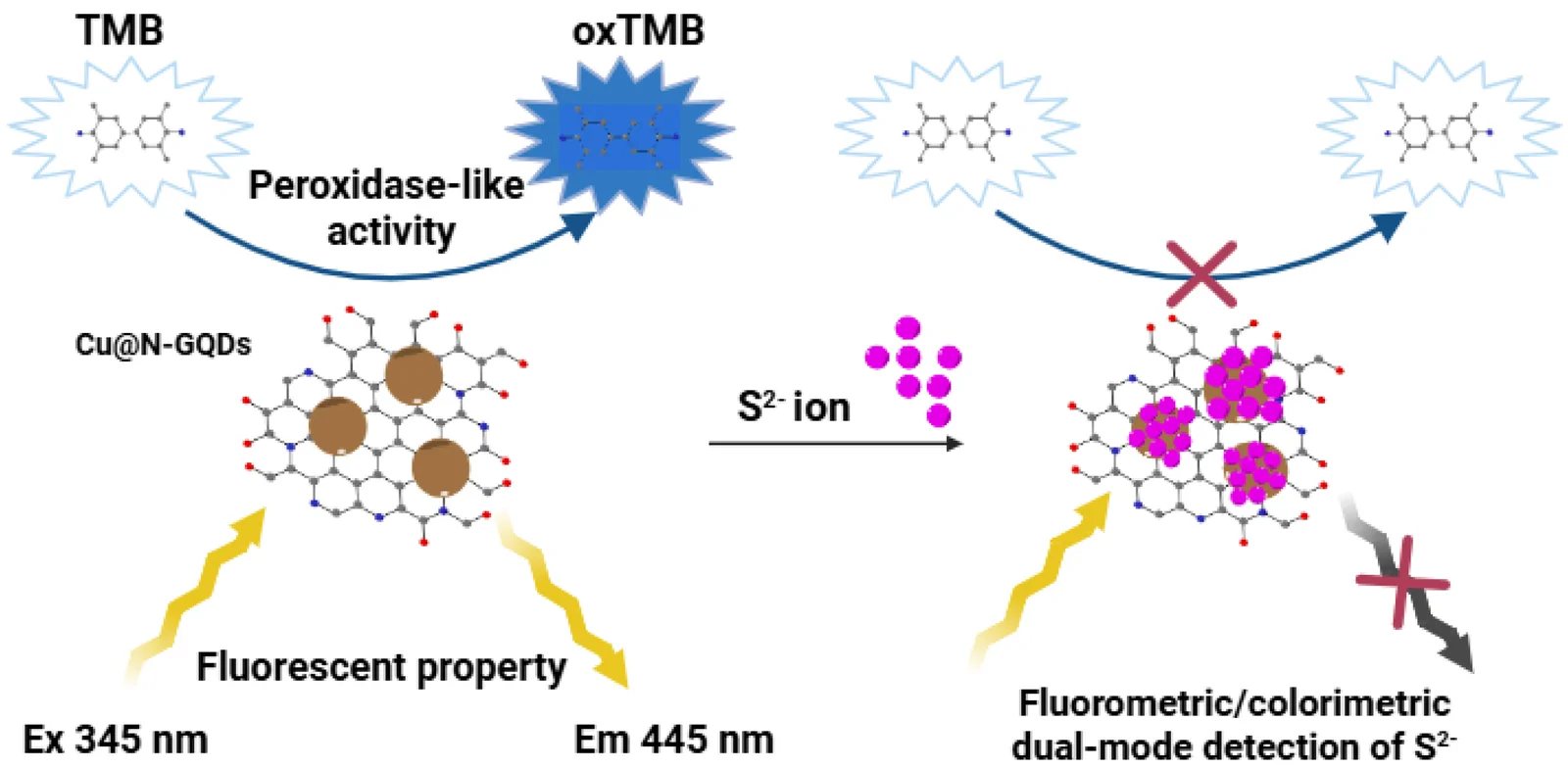
Schematic illustration of the dual-mode (fluorometric and colorimetric) sensing for sulfide ions using Cu@N-GQDs.

Cu@N-GQDs were synthesized through a one-step hydrothermal method that facilitated the in situ growth of copper oxide species on the surface of N-GQDs. During the reaction, citric acid served as a carbon precursor, while ammonium hydroxide provided both nitrogen doping and reductive conditions. Copper chloride provided the Cu^2+^ ions, which were reduced and stably anchored onto the surface of the forming N-GQDs during thermal treatment. This synthetic route yielded nanohybrids with both fluorescent and peroxidase-like catalytic functionality. The conceptual basis of this design was that Cu incorporation could endow N-GQDs with dual functionality: enhanced peroxidase-mimicking activity and retained fluorescence, both modulated upon selective interaction with sulfide ions (S^2−^). Sulfide ions, due to their high affinity for copper, were expected to bind with surface Cu species, simultaneously quenching fluorescence and suppressing catalytic activity in a concentration-dependent manner (Figure 1).

**Figure 2:**
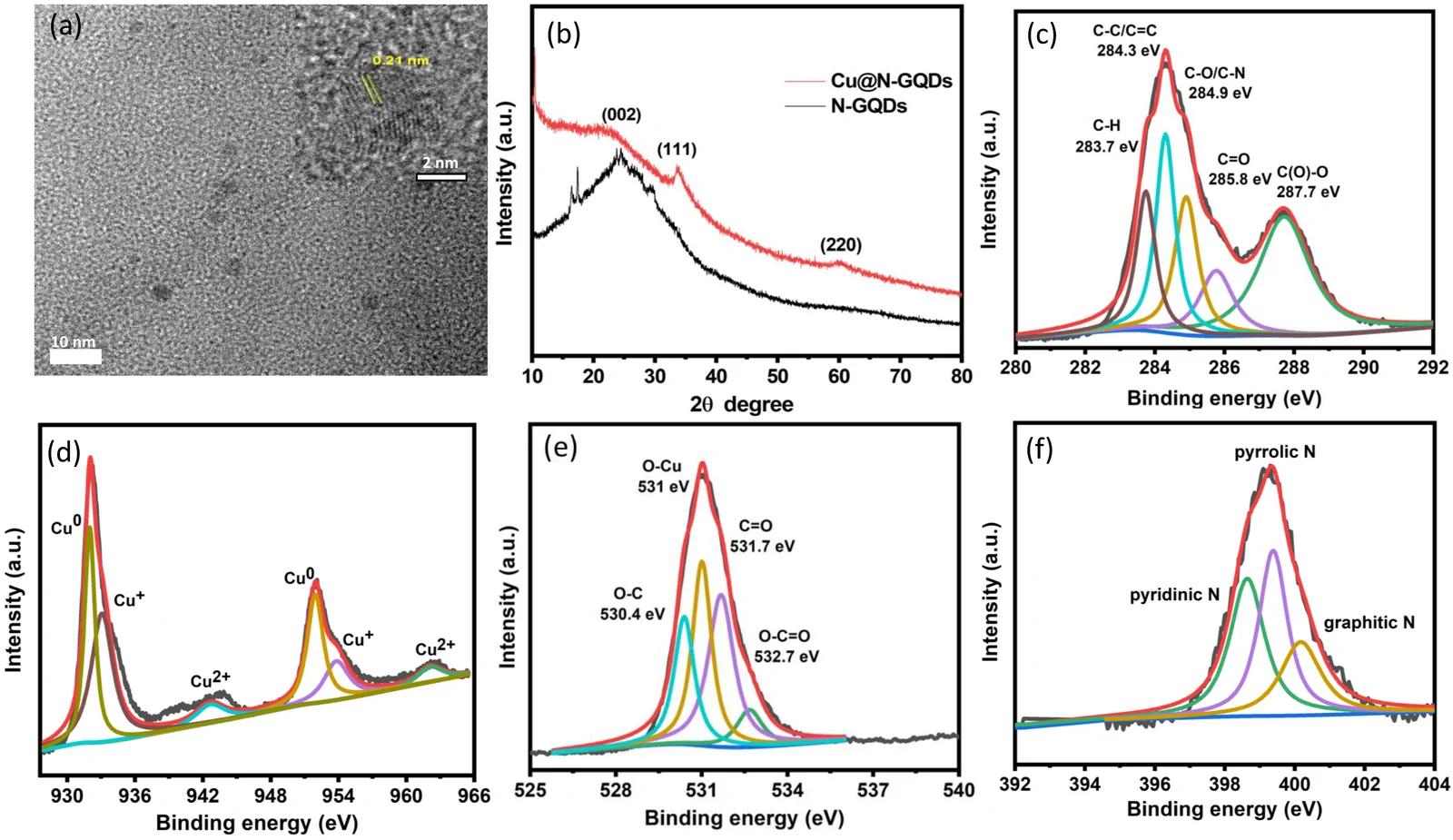
Characterization of Cu@N-GQDs. (**a**) TEM image with high-resolution TEM (inset) showing lattice fringes indicating the (100) plane of graphitic carbon. (**b**) XRD spectra showing the characteristic diffraction peaks of Cu_2_O and graphitic carbon. High-resolution XPS spectra of (**c**) C 1s, (**d**) Cu 2p, (**e**) O 1s, and (**f**) N 1s, confirming the presence and electronic states of C, Cu, O, and N.

TEM analysis showed that Cu@N-GQDs were spherical particles with an average diameter of 3.2 ± 0.4 nm (Figure 2a). Complementary DLS analysis was performed to determine the hydrodynamic size distribution of Cu@N-GQDs in dispersion (Figure S2). The average diameter was found to be 3.3 ± 0.1 nm, closely matching the size obtained from TEM measurements. The inset high-resolution TEM image revealed lattice fringes with a 0.21 nm spacing, consistent with the (100) plane of graphitic carbon [24], suggesting the crystallinity of the carbonaceous domain. XRD analysis displayed a broad diffraction peak at 2θ ≈ 24.5°, indicative of graphitic (002) planes, and additional peaks at 33.6° and 60.4° corresponding to the (111) and (220) planes of Cu_2_O, respectively (JCPDS No. 05-0667) [25,26] (Figure 2b). These results confirm the formation of a crystalline copper oxide phase embedded within a graphitic carbon matrix. XPS confirmed the elemental composition, with clear signals for C, N, O, and Cu (Figure S3a). High-resolution C 1s spectra featured peaks for C–H (283.7 eV), C–C/C=C (284.3 eV), C–O/C–N (284.9 eV), C=O (285.8 eV), and COOH (287.7 eV) [27,28] (Figure 2c). The Cu 2p spectrum displayed peaks at 932.1 and 952.2 eV, corresponding to Cu 2p_3/2_ and Cu 2p_1/2_ for Cu and Cu^+^, along with satellite peaks, confirming the presence of Cu(II) species [29] (Figure 2d). An O 1s peak at 531 eV was consistent with Cu_2_O lattice oxygen [29] (Figure 2e), while the N 1s spectrum showed pyridinic (398.7 eV), pyrrolic (399.4 eV), and graphitic N (400.2 eV) [30] (Figure 2f), verifying successful nitrogen doping. FT-IR spectra further supported the coordination of copper with surface functionalities. Compared to N-GQDs, Cu@N-GQDs exhibited diminished –OH stretching (~3328 cm^−1^), disappearance of the 1195 cm^−1^ O–H band, and changes in vibrational regions associated with COO^−^ and N-containing groups (1400–1600 cm^−1^) [31,32] (Figure S3b). These findings suggest that copper ions (Cu^2+^/Cu^+^) interact primarily with hydroxyl and amino groups during the composite formation.

### 3.2. Evaluation of Fluorescence and Peroxidase-Like Activity of Cu@N-GQDs

**Figure 3:**
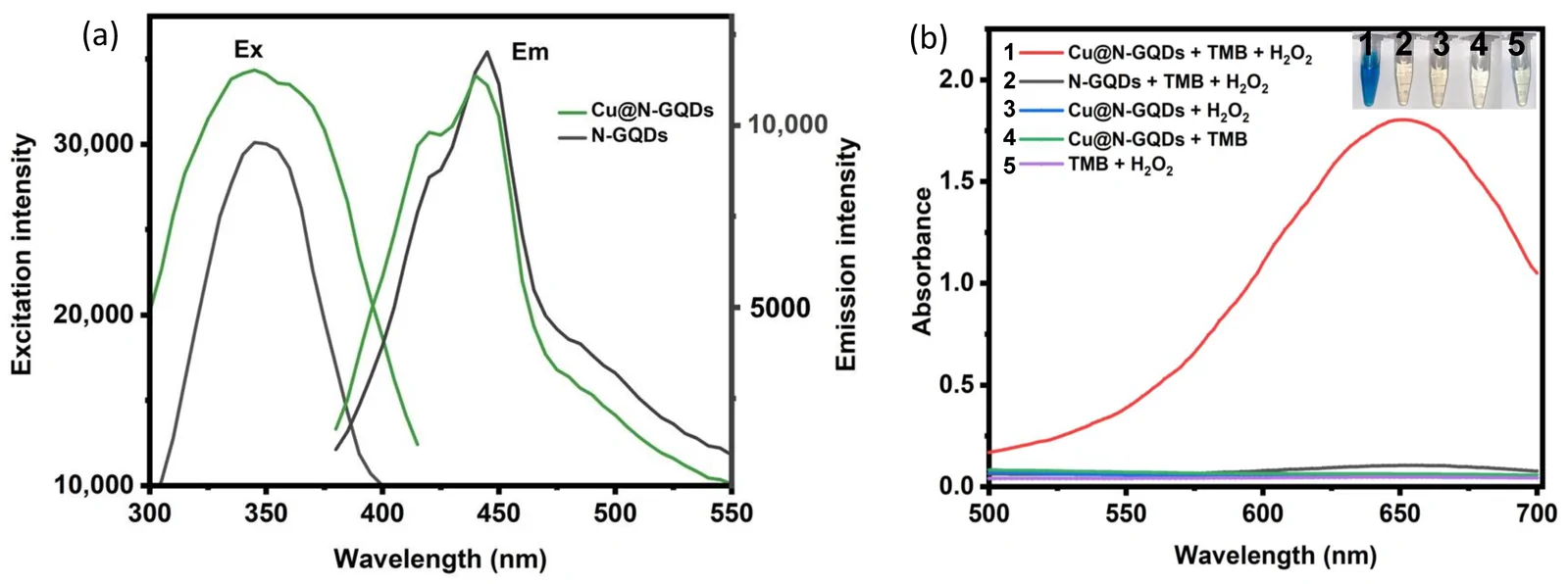
Evaluation of fluorescence and peroxidase-like activity of Cu@N-GQDs. (**a**) Comparison of fluorescence excitation and emission spectra of Cu@N-GQDs and N-GQDs. (**b**) Investigation of peroxidase-like activity of Cu@N-GQDs using TMB-H_2_O_2_ assay.

Fluorescent and peroxidase-mimetic dual-functionality of Cu@N-GQDs was examined by comparing it with that of N-GQDs. Both materials exhibited similar fluorescence centered at 445 nm (excitation wavelength = 345 nm), indicating that Cu anchoring did not disrupt the intrinsic fluorescence (Figure 3a). Peroxidase-like activity of N-GQDs was negligible, and importantly, Cu@N-GQDs exhibited significant activity to oxidize TMB in the presence of H_2_O_2_ (Figure 3b), indicating that surface copper species have a pronounced effect in achieving the catalytic activity. No significant color change was detected in the absence of H_2_O_2_, confirming negligible oxidase-like activity. The results demonstrated that Cu@N-GQDs have dual fluorescent and peroxidase-mimetic properties; the former is comparable and the latter is significantly enhanced compared with those of N-GQDs.

Peroxidase-like behaviors of Cu@N-GQDs were further investigated to elucidate their catalytic performance. Optimization studies showed that catalytic activity increased with Cu@N-GQD concentration and was saturated at ~0.1 mg/mL (Figure S4a). Time-dependent analysis demonstrated that the colorimetric signal was saturated after 15 min development (Figure S4b). The pH profile revealed peak activity at pH 5, which is similar with natural peroxidases [33] (Figure S4c). Maximum catalytic efficiency was achieved around RT, with performance declining at higher temperatures (Figure S4d). These results establish pH 5.0 and RT as optimal assay conditions. In addition, steady-state kinetics revealed Michaelis-Menten constants (K_m_) of 1.1 mM for TMB and 2.1 mM for H_2_O_2_ (Figure S5), which are comparable to other carbon dot-based peroxidase mimics [34,35,36].

Under the optimized assay conditions, the stability of Cu@N-GQD was systematically evaluated across a range of pH values and temperatures and compared with that of natural HRP. As shown in Figure S6, Cu@N-GQDs retained over 90% of their initial catalytic activity under all employed conditions, whereas HRP exhibited a sharp decline in activity, losing more than 50% below pH 3.0 and temperatures exceeding 50 °C. This marked improvement in stability highlights the robustness of Cu@N-GQDs under conditions where natural enzymes typically denature, underscoring their suitability for use in diverse and potentially harsh real-world environments.

### 3.3. Dual-Mode Optical Detection of Sulfide Ions

**Figure 4:**
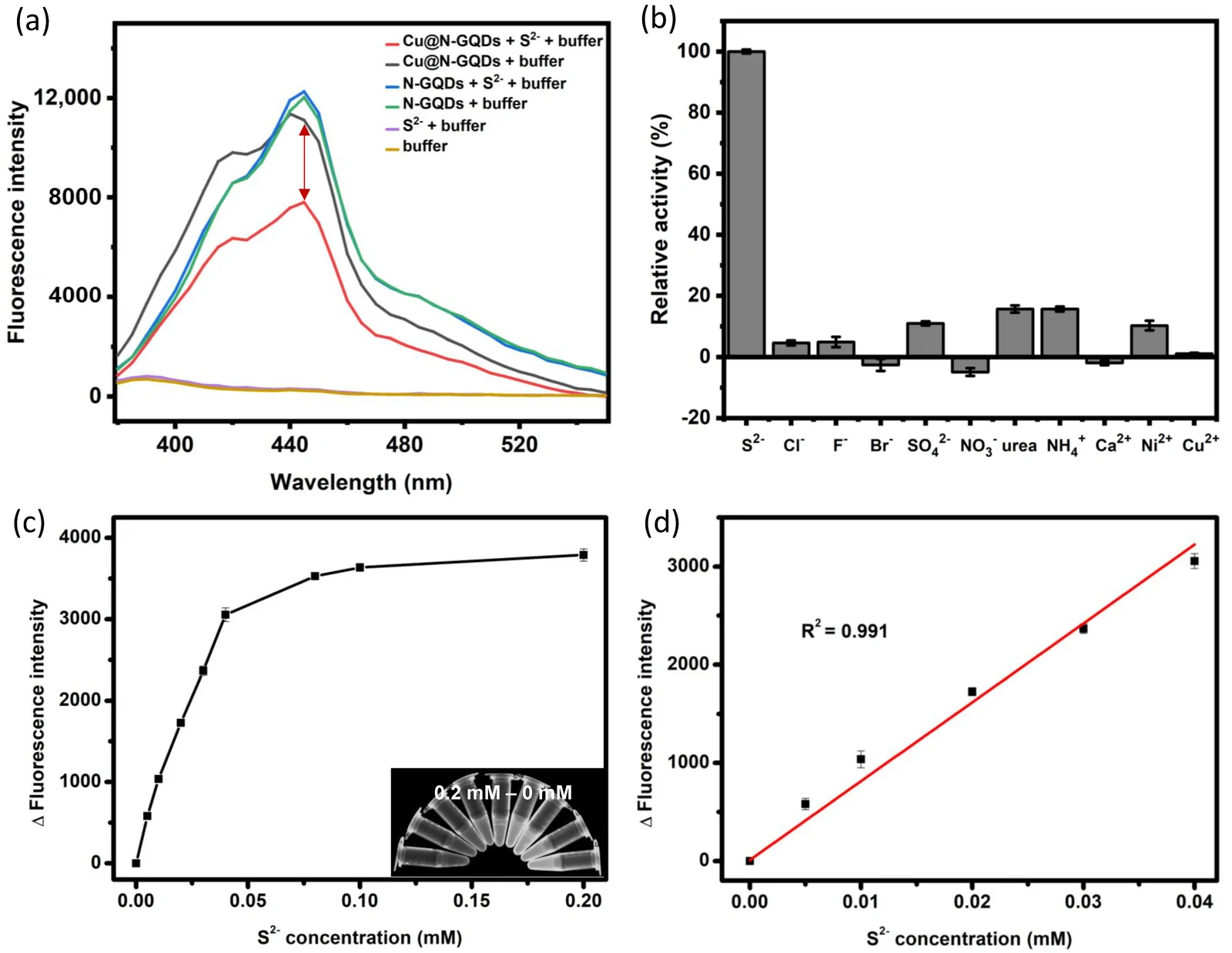
Fluorometric detection of sulfide ions using Cu@N-GQDs. (**a**) Fluorescence quenching in response to S^2−^. (**b**) Selectivity to detect S^2−^. Response to 100 µM S^2−^ compared with those of potential interfering species at 2 mM concentrations. (**c**) Dose-response curve with real fluorescent images, and (**d**) linear calibration plot for quantitative analysis of S^2−^.

The dual-mode sensing capability of Cu@N-GQDs was explored using Na_2_S as a S^2−^ source. In the fluorometric mode, the Cu@N-GQDs exhibited pronounced fluorescence quenching—up to ~30% reduction in emission intensity at 445 nm—upon the addition of S^2−^, while N-GQDs lacking surface copper species showed negligible response under identical conditions (Figure 4a). This selective quenching effect is attributed to the strong affinity between sulfide ions and surface-anchored Cu ions, resulting in the formation of Cu–S coordination complexes that perturb the local electronic environment and non-radiatively dissipate excited-state energy [37,38,39]. Notably, the quenching response was highly specific to sulfide ions. Control experiments demonstrated that no significant fluorescence change occurred in the presence of potentially interfering species, including common anions (Cl^−^, F^−^, Br^−^, SO_4_^2−^, and NO_3_^−^), cations (NH_4_^+^, Ca^2+^, Ni^2+^, and Cu^2+^), and small molecules such as urea, even when tested at 20-fold higher concentrations (Figure 4b and Figure S7). This high specificity is likely due to the unique thermodynamic favorability of Cu–S complexation, which forms highly insoluble CuS (the solubility product constant K_sp_ ≈ 10^−36^) even at low concentrations [40], unlike other inorganic ions.

Quantitatively, the fluorescence intensity showed a strong linear correlation with S^2−^ concentration in the range of 0–40 µM (R^2^ = 0.991), with a calculated limit of detection (LOD) of 0.5 µM (Figure 4c,d). This detection threshold ranks favorably among existing nanomaterial-based optical sulfide sensors, placing the Cu@N-GQD platform among the most sensitive dual-mode systems reported to date [13,17,22,41,42,43] (Table 1).

**Figure 5:**
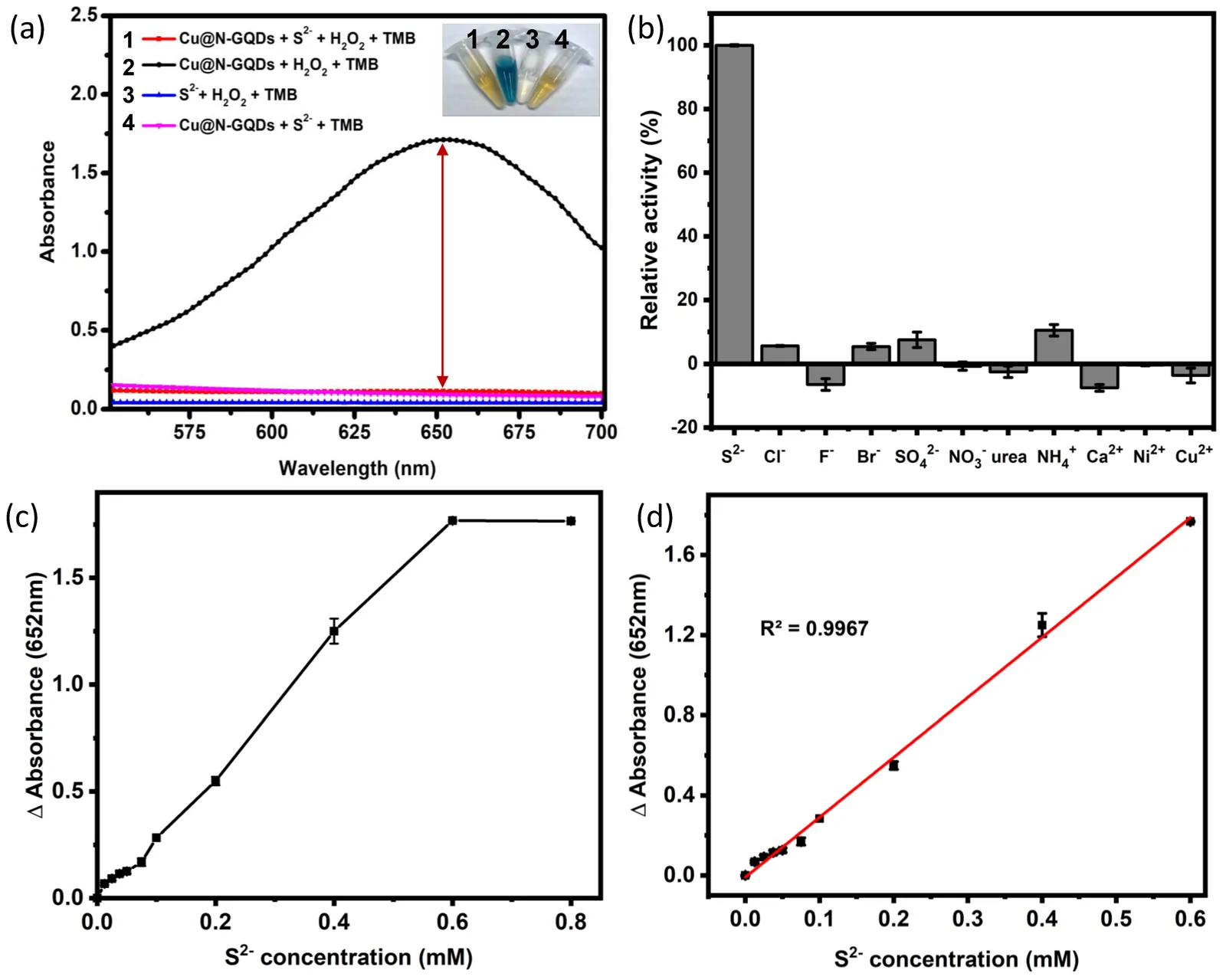
Colorimetric detection of sulfide ions using Cu@N-GQDs. (**a**) Suppression of TMB oxidation in presence of S^2−^. (**b**) Selectivity to detect S^2−^. Response to 100 µM S^2−^ compared with those of potential interfering species at 2 mM concentrations. (**c**) Dose-response curve, and (**d**) linear calibration plot for quantitative analysis of S^2−^.

In parallel, the colorimetric detection mode was based on the Cu@N-GQDs’ peroxidase-like activity, which catalyzes the oxidation of TMB to a blue-colored product (absorbance at 652 nm) in the presence of H_2_O_2_. Upon exposure to S^2−^, this catalytic activity was markedly inhibited (Figure 5a), presumably due to surface Cu centers being passivated by sulfide ions through Cu–S bonding. This complexation likely deactivates redox-active sites and impairs the electron transfer required for TMB oxidation. The sensor exhibited strong specificity in this mode as well, with no significant signal suppression observed for interfering substances (Figure 5b). A clear linear response was observed in the 0–600 µM S^2−^ range, with an LOD of 3.5 µM (Figure 5c,d). The visually discernible color change, from deep blue to pale, makes this mode well-suited for portable, instrument-free detection in field settings.

Finally, the applicability of the Cu@N-GQD platform for real sample analysis was assessed using tap water spiked with various concentrations of S^2−^. In both detection modes, the sensor provided accurate and precise measurements with recoveries ranging from 95.4–104.6% (fluorescence) to 95.2–103.3% (colorimetry), and coefficients of variation below 3.3% and 1.5%, respectively (Table 2). The developed Cu@N-GQD-based sensor also showed excellent selectivity toward target S^2−^ using tap water spiked with various interfering molecules (Figure S8). These findings affirm the reliability, selectivity, and dual-mode adaptability of Cu@N-GQDs, highlighting their strong potential as a cost-effective and field-deployable sensing platform for environmental sulfide monitoring.

## 4. Conclusions

We successfully developed Cu@N-GQDs as bifunctional nanozymes for dual-mode optical detection of sulfide ions. The Cu@N-GQDs exhibited both strong intrinsic fluorescence and robust peroxidase-like activity, enabled by the in situ growth of copper oxide species on N-GQDs. Upon interaction with sulfide ions, these nanocomposites showed simultaneous fluorescence quenching and catalytic inhibition via Cu-S complexation, allowing complementary dual optical readouts. The system achieved high sensitivity and excellent selectivity against common interfering species. Moreover, the sensor demonstrated reliable recovery and precision in real tap water samples without requiring sophisticated instrumentation. These findings underscore the strong potential of Cu@N-GQDs as a versatile dual-mode optical sensing platform for the precise and sensitive detection of toxic sulfide ions. Looking ahead, this work lays the foundation for the development of next-generation, field-deployable analytical devices, with future efforts directed toward integrating the system into portable microfluidic devices, paper-based diagnostics, and wearable sensor formats for on-site environmental and biomedical monitoring.

## Figures and Tables

**Table 1:** Comparison of linear range and LOD values for sulfide ion detection using our Cu@N-GQD-based biosensor and those of optical sensing systems reported previously.

Catalytic Materials	Method	Linear Range (μM)	LOD (μM)	Reference
Mn-doped ZnS QDs	Fluorometric	1.2–26	0.33	[41]
PDA@Co_3_O_4_	Fluorometric	<200	4.3	[22]
DSP-Based Assay	Colorimetric Fluorometric	0.5–40 0.5–20	2.17 2.5	[13]
AuNCs and CDs	Colorimetric Fluorometric	<10 1–50	4.0 0.35	[42]
Ag^+^ and CDs	Fluorometric	1–100	0.43	[17]
3-Mercaptopropionic acid functionalized CdS QDs	Fluorometric	3.1–55.7	6.5	[43]
Cu@N-GQDs	Colorimetric Fluorometric	10–600 2–40	3.5 0.5	This work

**Table 2:** Accuracy and precision of the Cu@N-GQD-based dual-mode biosensor for quantifying sulfide ions spiked in real tap water samples.

Method	Spiked Level (µM)	Measured ^a^ (µM)	Recovery ^b^ (%) (*n* = 3)	CV ^c^ (%)
Fluorescence	25	24.3	97.3	2.2
	50	52.5	104.6	3.3
	100	95.4	95.4	2.2
Colorimetry	50	47.7	95.2	1.5
	100	99.8	99.7	1.3
	150	155.5	103.3	1.4

^a^ Mean value of three independent measurements. ^b^ Measured value/expected value × 100. ^c^ Coefficient of variation (CV) = (SD/mean) × 100.

## Data Availability

The original contributions presented in this study are included in the article/supplementary material. Further inquiries can be directed to the corresponding author.

## Supplementary Materials

The following supporting information can be downloaded at: https://www.mdpi.com/article/10.3390/bios15080528/s1, Table S1: Assay conditions for measuring the peroxidase-like activity of Cu@N-GQDs; Figure S1: Schematic illustration of one-pot hydrothermal synthesis of Cu@N-GQDs; Figure S2: Hydrodynamic size distribution of Cu@N-GQDs measured by DLS; Figure S3: (**a**) XPS spectra and elemental ratios of Cu@N-GQDs and (**b**) FT-IR spectra of N-GQDs and Cu@N-GQDs; Figure S4: Effects of (**a**) Cu@N-GQDs concentration, (**b**) assay time, (**c**) pH, and (**d**) temperature on the peroxidase-like activity of Cu@N-GQDs; Figure S5: Michaelis-Menten curves and corresponding Lineweaver-Burk plots (inset) of Cu@N-GQDs toward (**a**) TMB and (**b**) H_2_O_2_ (n = 3); Figure S6: Comparison of stabilities of Cu@N-GQDs and horseradish peroxidase (HRP) in ranges of (**a**) pH and (**b**) temperature; Figure S7: Real fluorescent images for showing selectivity to detect sulfide ions using Cu@N-GQD-based biosensor; Figure S8: Selectivity to detect S^2−^ (100 μM) in tap water spiked with various potentially interfering molecules (2 mM) by (**a**) fluorometric and (**b**) colorimetric methods.
