# Supplementary material for: Dual-Mode Optical Detection of Sulfide Ions Using Copper-Anchored Nitrogen-Doped Graphene Quantum Dot Nanozymes
Source: Biosensors (Basel). 2025 Aug 13;15(8):528. doi: 10.3390/bios15080528 (PMC12384640; doi:10.3390/bios15080528)
Supplement: Supplementary file 1 [file biosensors-15-00528-s001.zip › biosensors-3794562-supplementary.pdf]

biosensors

MDPI

Supplementary Materials

Dual-mode optical detection of sulfide ions using copper-anchored nitrogen-doped graphene quantum dot nanozymes

Van Anh Ngoc Nguyen, Trung Hieu Vu, Phuong Thy Nguyen and Moon Il Kim *

Department of BioNano Technology, Gachon University, 1342 Seongnamdae-ro, Sujeong-gu, Seongnam, Gyeonggi 13120, Republic of Korea; vananh.ngocng@gmail.com (V.A.N.N.); hieu.vutrung24596@gmail.com (T.H.V.); nnphuongthy18@gmail.com (P.T.N.)
* Correspondence: moonil@gachon.ac.kr (M.I.K.); Tel.: +82-31-750-8563

Table S1. Assay conditions for measuring the peroxidase-like activity of Cu@N-GQDs.

Assay conditions	Employed values
Cu@N-GQDs concentration	100 μg/mL
TMB concentration	0.5 mM
H_2O_2 concentration	10 mM
Buffer and pH	50 mM HEPES, pH 5.0
Incubation time	15 min
Temperature	Room temperature (22 °C)

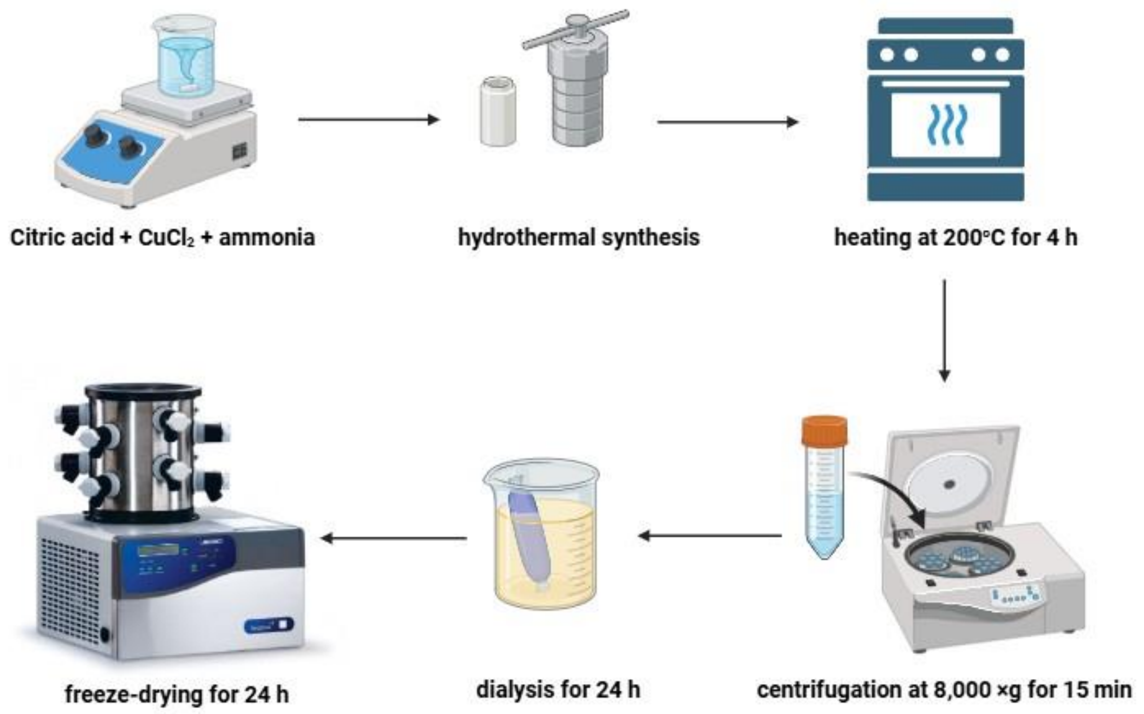

Figure S1. Schematic illustration of one-pot hydrothermal synthesis of Cu@N-GQDs.

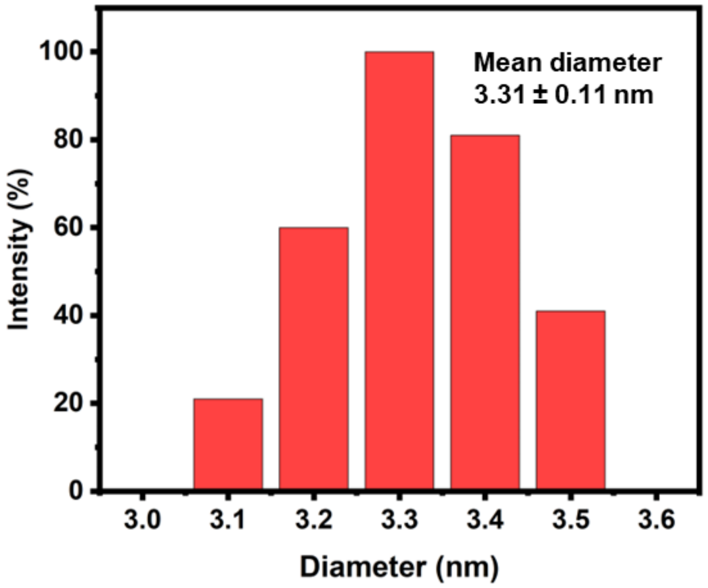

Figure S2. Hydrodynamic size distribution of Cu@N-GQDs measured by DLS.

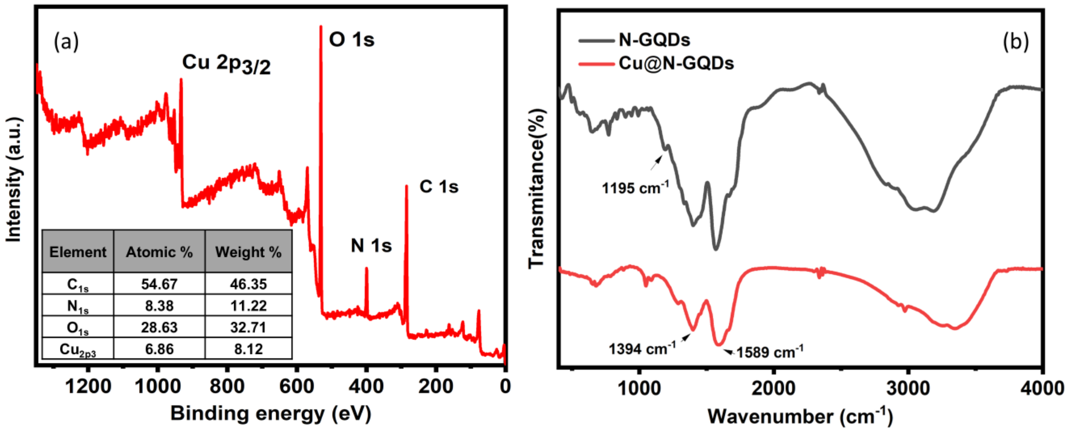

Figure S3. (**a**) XPS spectra and elemental ratios of Cu@N-GQDs and (**b**) FT-IR spectra of N-GQDs and Cu@N-GQDs.

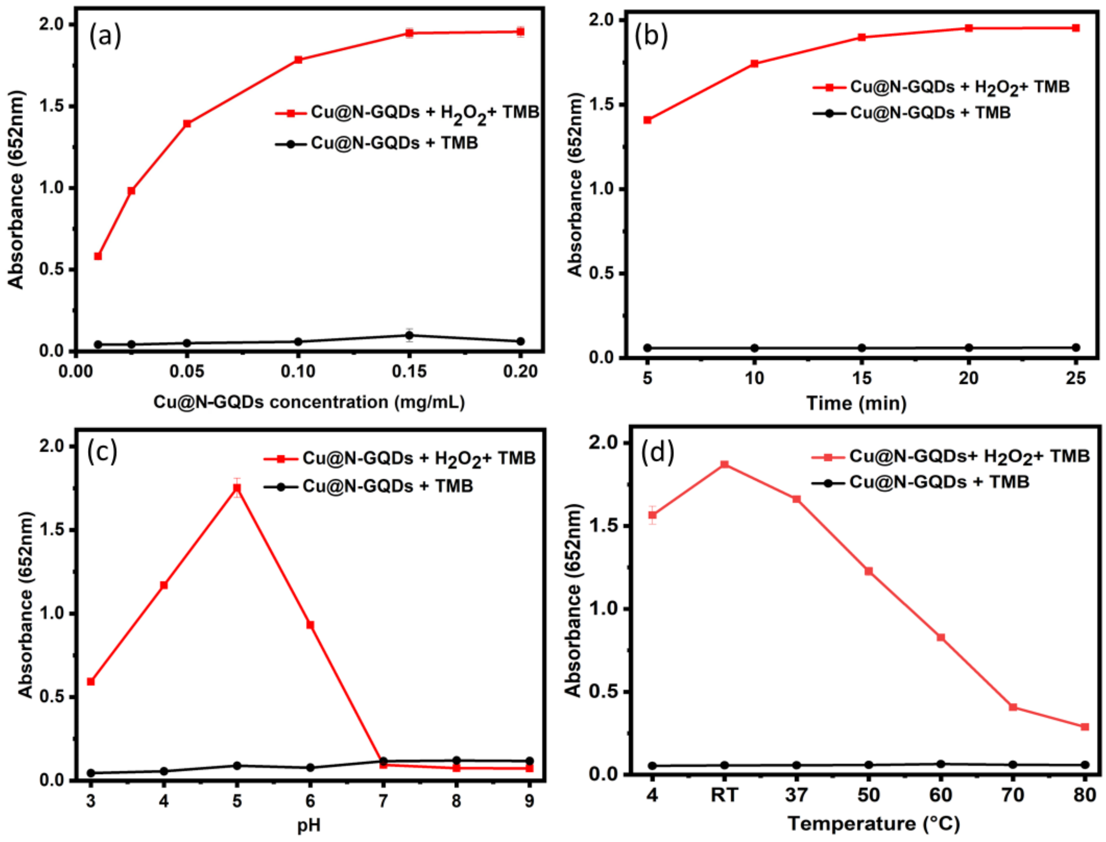

Figure S4. Effects of (**a**) Cu@N-GQDs concentration, (**b**) assay time, (**c**) pH, and (**d**) temperature on the peroxidase-like activity of Cu@N-GQDs.

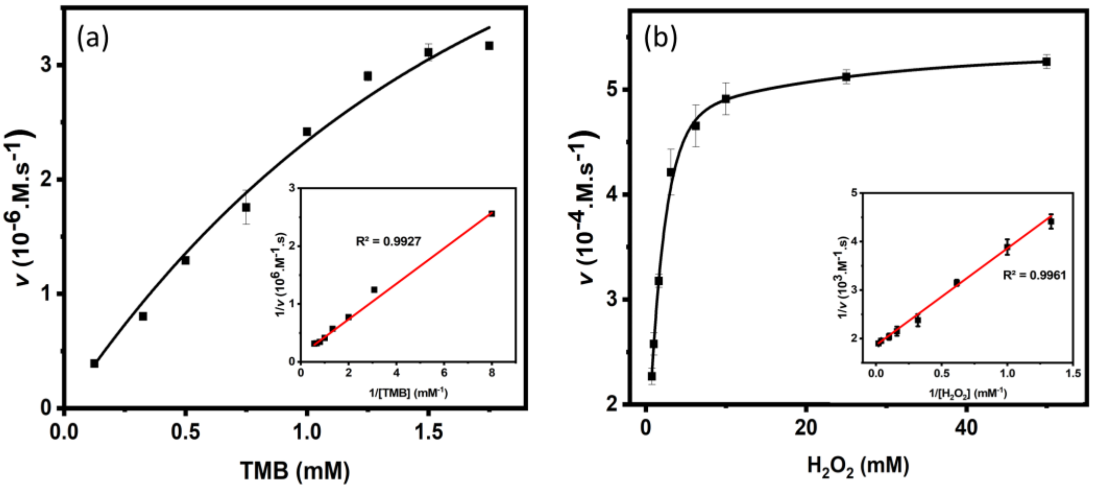

Figure S5. Michaelis-Menten curves and corresponding Lineweaver-Burk plots (inset) of Cu@N-GQDs toward (**a**) TMB and (**b**) H_2O_2 (n = 3).

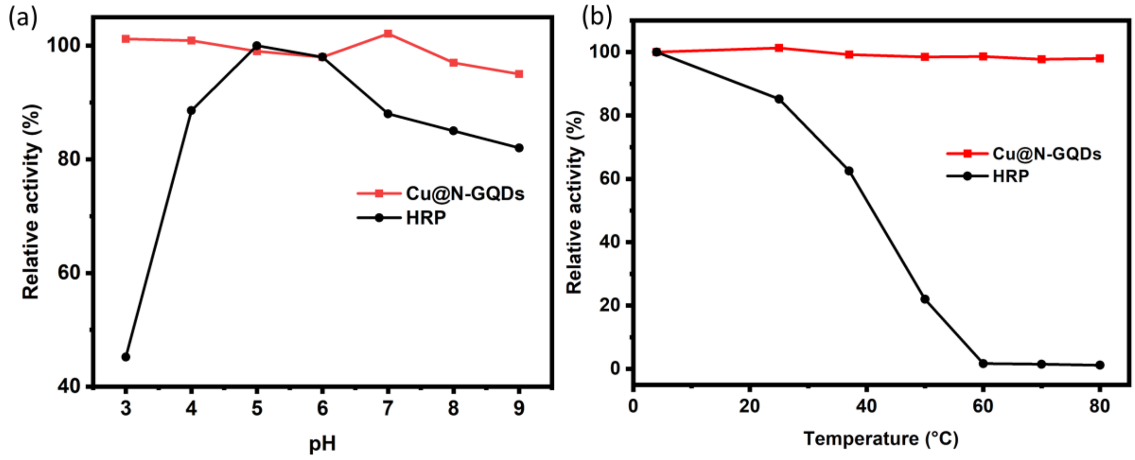

Figure S6. Comparison of stabilities of Cu@N-GQDs and horseradish peroxidase (HRP) in ranges of (**a**) pH and (**b**) temperature.

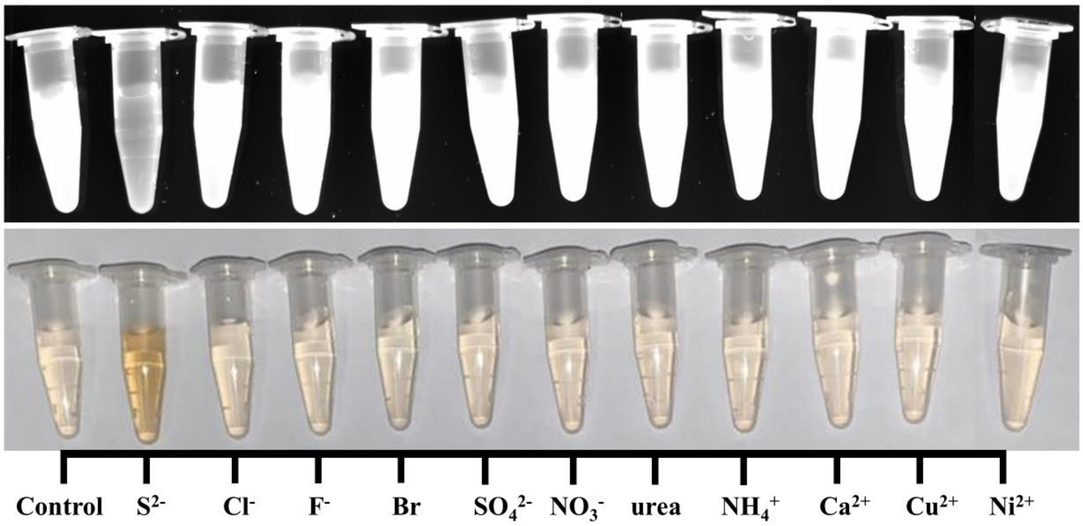

Figure S7. Real fluorescent images for showing selectivity to detect sulfide ions using Cu@N-GQD-based biosensor.

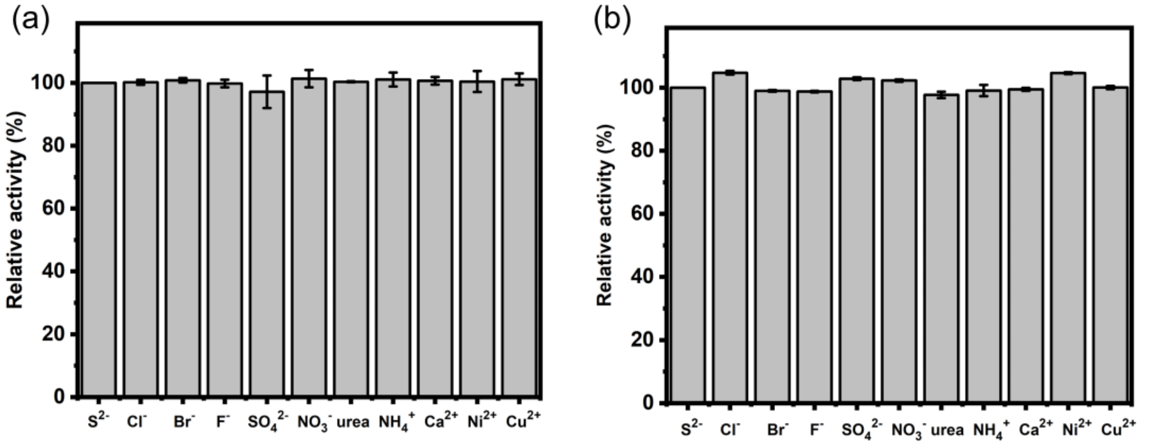

Figure S8. Selectivity to detect S^{2-} (100 μM) in tap water spiked with various potentially interfering molecules (2 mM) by (**a**) fluorometric and (**b**) colorimetric methods.

© 2025 by the authors. Submitted for possible open access publication under the terms and conditions of the Creative Commons Attribution (CC BY) license (http://creativecommons.org/licenses/by/4.0/).
